# Correlation between Microstructure and Magnetism in Ball-Milled SmCo_5_/α-Fe (5%wt. α-Fe) Nanocomposite Magnets

**DOI:** 10.3390/ma14040805

**Published:** 2021-02-08

**Authors:** Anna Bajorek, Paweł Łopadczak, Krystian Prusik, Maciej Zubko

**Affiliations:** 1 A. Chełkowski Institute of Physics, University of Silesia in Katowice, 75 Pułku Piechoty 1, 41-500 Chorzów, Poland; p.lopadczak@gmail.com; 2 Silesian Center for Education and Interdisciplinary Research, University of Silesia in Katowice, 75 Pułku Piechoty 1A, 41-500 Chorzów, Poland; krystian.prusik@us.edu.pl (K.P.); maciej.zubko@us.edu.pl (M.Z.); 3 Institute of Materials Science, University of Silesia in Katowice, 75 Pułku Piechoty 1A, 41-500 Chorzów, Poland

**Keywords:** rare-earth intermetallics, high-energy ball milling, soft/hard magnetic nanocomposites, permanent magnets, microstructure, magnetic properties, exchange coupling, electronic structure

## Abstract

Magnetic nanocomposites SmCo_5_/α-Fe were synthesized mechanically by high-energy ball milling (HEBM) from SmCo_5_ and 5%wt. of α-Fe powders. The X-ray diffraction analysis reveals the hexagonal 1:5 phase as the main one accompanied by the cubic α-Fe phase and 2:17 rhombohedral as the secondary phase. The content of each detected phase is modified throughout the synthesis duration. A significant decrease in crystallite size with a simultaneous increase in lattice straining is observed. A simultaneous gradual reduction in particle size is noted from the microstructural analysis. Magnetic properties reveal non-linear modification of magnetic parameters associated with the strength of the exchange coupling induced by various duration times of mechanical synthesis. The highest value of the maximum energy product *(BH)_max_* at room temperature is estimated for samples milled for 1 and 6 h. The intermediate mixed-valence state of Sm ions is confirmed by electronic structure analysis. An increase in the Co magnetic moment versus the milling time is evidenced based on the performed fitting of the Co3*s* core level lines.

## 1. Introduction

Nanocomposite permanent magnets have been intensively studied for about two decades due to their possible application in eco-sustainable and energy-saving technologies, leading towards device miniaturization [1,2,3]. Among a variety of synthesis approaches, substantial progress has been achieved in the preparation of surfactant-assisted high-energy ball-milled (SA-HEBM) nanomagnets as a first step in the production of exchange-coupled metastable permanent magnets [1,2,3]. Most of all, high-energy ball milling (HEBM) allows for controlling the micro/nanostructure of synthesized materials which is often directly related to the enhancement of coercivity (*H*_C_) or the maximum energy product *(BH)_max_*. As it has been shown, such relation is fulfilled in many nanostructured R-T-based intermetallics, e.g., Sm–Co [4,5,6,7,8,9,10,11,12,13,14,15,16,17,18,19,20,21,22,23], Pr–Co [24,25,26,27,28] and Nd–Fe–B nanopowders [29,30,31] and some others having a 1:3 rhombohedral crystal structure [24,25,26,32,33,34,35,36,37,38,39,40,41]. Nonetheless, as it has been evidenced that SmCo_5_-based nanomagnets are recognized as materials with the highest coercivity and Curie temperature [1,2]. Thus, optimization within the synthesis of nanocomposites based on hard SmCo_5_ and soft Fe/Co phases, in which enhanced magnetic parameters are expected, is a highly demanded challenging task. The selection of HEBM parameters accompanied by the quantity of iron as a soft magnetic component is a crucial factor in designing such materials as exchange-coupled nanomagnets.

So far, SmCo_5_-based nanocomposites have been synthesized in an argon atmosphere or by the use of the wet SA-HEBM procedure, usually applied for up to 10 h, and also by magnetic field-assisted HEBM [13,42,43,44,45,46,47,48,49,50,51,52,53,54,55,56,57,58]. The comparison of properties achieved for magnetic field-milled SmCo_5_/α-Fe nanocomposite powders with 10 wt.% of α-Fe made by Saravanam et al. [13,46] with and without surfactants emphasizes the influential role of wet milling [46]. Although, in both cases, grain size reduction was observed in the early stages of milling, with the addition of surfactants, the coercivity and anisotropy of as-milled powders were enhanced due to a decrease in particle aggregation and trapping of the fine particles. In addition, surfactant-assisted milling enables the possible grain orientation and prevents an excessive formation of the α-Fe(Co) soft magnetic phase.

The microstructural studies performed so far, e.g., by Saravanam et al. [13] for 10 wt.% of α-Fe, revealed the platelet-like microstructure of the 1:5 phase with elongated α-Fe grains (grains size of 10 μm) embedded in the hcp-Sm(Co,Fe)_5_ matrix. In addition, the occurrence of the mechanically activated diffusion process leading to Fe/Co intermixing was evidenced as diffusion of Fe atoms in the SmCo_5_ phase and Co atoms in the α-Fe phase. Such phenomena were noted for 10–30 wt.% of α-Fe-based materials [13,40,47,48,49,51,52,53,54,55,56,57,58]. The interdiffusion process might be controlled by the annealing procedure, which is also reflected in the increase in grain size [13].

As it was shown, e.g., by Le Breton et al. [40] and Larde et al. [47], the applied heat treatment for as-milled nanocomposites stabilizes the formation of Sm(Co,Fe)_5_ and α-Fe(Co) phases with a gradual increase in the Fe content in the SmCo_5_ phase and a simultaneous increase in the Co content in the α-Fe phase dependent on the annealing temperature. Larde et al. [47], for a synthesized SmCo_5_/α-Fe (20 wt.% α-Fe) sample, described the nanosized α-Fe(Co) clusters as a core−shell structure with a Fe-rich core and a shell acting as the graded interface region. Additionally, the Co content in the core of clusters was dependent on their size. Thus, for the smaller cluster size, the Co content in its core was higher and vice versa. As it was demonstrated, the applied annealing process was reflected in a mean concentration of 55 ± 1% Fe and 45 ± 1% Co in the cores, while the Fe content in the matrix reached 17 ± 2%. Thus, the microstructural analysis exhibited a non-homogeneous distribution of soft α-Fe-rich regions which, in the major part, were composed of spheroidal nanosized Fe(Co) clusters with a diameter of <20 nm accompanied by a few Fe-rich lamellae with several hundred nanometers in length and a thickness in the 20–100 nm range. Such an inhomogeneous distribution was reflected in the coupling of the soft and hard phases. Indeed, most of the soft-phase crystallites with smaller size were well coupled to the hard phase, but those with the largest size demonstrated the worst coupling. The performed Monte Carlo simulations confirmed that the Fe/Co interdiffusion can lead to an increase in the coercive field.

The magnetic properties of as-milled nanocomposites may be enhanced by further processing of synthesized powders. So far, two methods have been applied to SmCo_5_/α-Fe, namely, spark plasma sintering (SPS) [45,51,53] and plasma pressure consolidation (PPC) [50]. The combination of SPS/PPC parameters with precedents of the HEBM parameters has to be carefully planned in order to achieve optimized magnetic properties.

After applying the first method mentioned, the increase in coercivity is noted as in the case of, e.g., SmCo_5_/α-Fe (20 wt.% α-Fe) from *H*_C_ = 3.5 Oe (as-milled grains) to *H*_c_ = 8.2 kOe, reasonably combined with the maximum energy product of (*BH*)*_max_* = 278.7 kJ/m^3^ (sintered powders) [45]. In comparison, Pop et al. [51] achieved the highest value of the maximum energy product *(BH)_max_* of 84 kJ/m^3^ for a SmCo_5_/α-Fe (20 wt.% α-Fe) sample milled for 8 h and annealed at 550 °C for 1.5 h. The enhancement of magnetic parameters might be controlled by the amount of SmCo_5_ powder in composites and simultaneously by SPS parameters. As it was found by Saito et al. [51] that SmCo_5_/α-Fe (20 wt.% α-Fe) magnets produced at 873 K under a vacuum and a pressure of 100 MPa composed of Fe and SmCo_5_ phases exhibit a high remanence of 83.1 Am^2^/kg with a high coercivity of 0.98 MA/m. The increase in SPS temperature to 973 K leads to a separation of the additional 2:17 phase which reduces coercivity. A relatively significant coercivity was denoted by Rama Rao et al. [53], who found a maximum coercivity of 8.9 kOe in SmCo_5_/α-Fe (5 wt.% α-Fe) nanocomposite magnets (a combination of 10 h of milling, consolidation under 2 T magnetic field, SPS in a vacuum at the sintering temperature 700–740 °C and pressure 10–10.5 kN for 5 min) due to stronger exchange coupling between the hard and soft magnetic phases than evidenced for the 10 wt.% α-Fe-containing sample. Nonetheless, in the synthesized samples, besides the main 1:5 phase, other phases of 2:17, 1:2 and Fe(Co) having grains in the range of 50–100 nm were found [54].

PPC was found by Zheng et al. [50] as a promising second step in the production of bulk composite magnets made of SmCo_5_/α-Fe (20 wt.% α-Fe) with high density at reduced temperature. The first step in such synthesis was low-energy ball milling and aligning the as-milled specimen in the magnetic field of 1.2 T for 5 min. As it was shown, the magnetic coupling between hard and soft phases is maintained after PPC despite the evident soft phase grain growth seemingly beyond the theoretically predicted limits. The authors also demonstrated the formation of the 2:17 phase due to a limitation in the Fe solubility in the SmCo_5_ matrix. The microstructure of the synthesized nanocomposite was detected as uniformly CoFe-coated hard Sm–Co particles.

Therefore, as it was demonstrated, mechanically milled specimens may be successfully considered as a starting material in the fabrication of anisotropic nanocomposite magnets with enhanced magnetism. In addition, the variety of technical parameters in SmCo_5_/α-Fe nanocomposite synthesis leads to the ability to control the magnetic parameters by tuning the exchange coupling between soft and hard phases. One of the adjusted parameters is definitely the amount of the α-Fe phase. In our paper, we focused on its low content (5%wt.) as it has been the least analyzed so far. Our study aims to bring out a detailed investigation based on the synthesis of SmCo_5_/α-Fe nanocomposite nanopowders via modified HEBM. We analyze the microstructure and magnetism in as-milled powders as a function of the applied milling time. In addition, we present the modification within the electronic structure versus milling, which for the SmCo_5_/α-Fe-based nanocomposites is undoubtedly a novelty element.

## 2. Materials and Methods

The SmCo_5_ compound was prepared by arc melting from high-purity elements under argon atmosphere. The ingot was melted several times in order to obtain homogeneity. Afterwards, the as-cast sample was wrapped in tantalum foil, placed in quartz tubes and annealed at 24 h at 1100 °C. The crystal structure was checked by means of X-ray powder diffraction (XRD) using an Empyrean PANalytical (Malvern Instruments, Malvern, UK) diffractometer equipped with a Cu X-ray source (Kα_1_ of 1.54056 Å). In order to obtain the SmCo_5_ nanopowder, first, the bulk compound was crushed and pre-milled with a standard agate mortar for about 10 min. Afterwards, the mixture of SmCo_5_ nanopowder and 5%wt of α-Fe was ground for 0.5, 1, 1.5, 2, 4, 6, 8 and 10 h with a 30 Hz frequency (1800 rpm) using a Mixer Mill 400 (Retsch, Haan, Germany). As in the case of previously synthesized intermetallics [16,17,34,35,36,37,38,39,40,41], an innovative wet milling method was carried out in dimethylformamide (DMF) using Eppendorf vials and 2 mm ZrO_2_ balls with a balls to powder ratio of about 10:1. The structure and morphology of the as-milled specimens were investigated by high-resolution scanning electron microscopy (HR-SEM) on a JEOL JSM-7100F FEG (field emission gun) operated at 15 kV in secondary electron (SE) mode and X-ray powder diffraction (XRD) (Empyrean PANalytical, Cu X-ray source Kα_1_ of 1.54056 Å). The XRD studies were carried out at room temperature using the solution containing the mixture of SmCo_5_/α-Fe nanopowders and DMF dried on the circular quartz plate.

The SEM images were recorded after each stage of grinding taken in the SE mode. All examined powders were fixed to the sample holder by the carbon double-coated conductive tape. The EDXS composition elemental maps were collected using JEOL EDX SSD and IXRF LN detectors attached to the JEOL JSM 7100F and JSM 6480 SEM (JEOL Ltd., Tokyo, Japan), respectively.

After the last stage of shredding (after 10 h), transmission electron microscopy (TEM) with a JEOL JEM 3010 (JEOL Ltd., Tokyo, Japan) was conducted. For TEM analysis, the sample was dried and purified in ethanol in the ultrasonic washer for 2 h in order to remove the excess DMF and to fracture the agglomerates. Afterwards, a few drops of the powder suspended in ethanol were attached adhesively to the carbon-coated Cu grid (400 mesh). The X-ray photoelectron spectroscopy (XPS) measurements with a PHI 5700/660 spectrometer (Physical Electronics Inc., Eden Prairie, MN, USA) were performed for samples milled for 0.5, 4 and 10 h. The XPS data were collected at room temperature using a monochromatic Al K_α_ (1486.6 eV) X-ray source. Powders were compacted and fixed to the sample holder by using the carbon double-coated conductive tape. Subsequently, such prepared specimen was measured in three separated steps. At first, the as-milled material was studied after its storage under ultra-high vacuum for about one week. Afterwards, ion etching by an Ar^+^ beam was applied for 1 h, and then the same specimen was measured. All the obtained spectra were calibrated using the C1s peak (EB = 284.8 eV) as an adventitious carbon usually accumulated on the surface of the sample and used as a reference for a charge correction.

The hysteresis loops were determined at 2 and 200 K based on the wide-range superconducting quantum interference device (SQUID) magnetometer MPMS XL7 (Quantum Design Inc., San Diego, CA, USA) under the change in the external magnetic field up to μ_0_H = 7 T.

## 3. Results and Discussion

### 3.1. XRD

**Figure 1:**
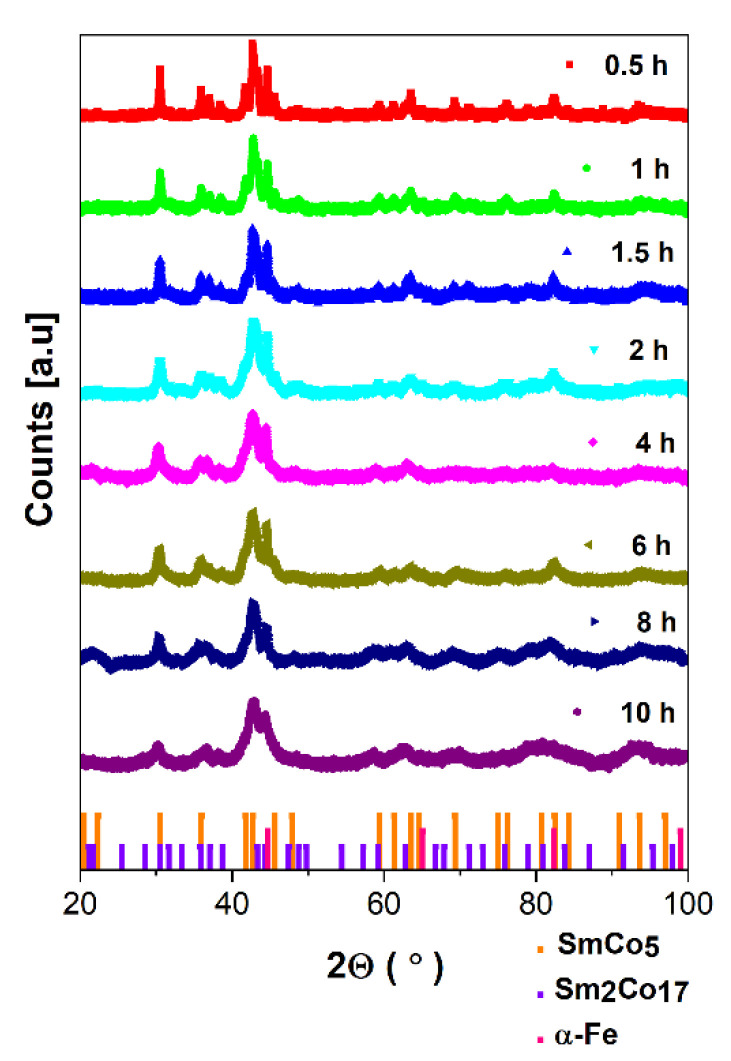
The evolution of XRD patterns for SmCo_5_/α-Fe nanocomposites (5%wt. of α-Fe) versus milling time. The detected crystal phases are denoted by bars: SmCo_5_—orange, Sm_2_Co_17_—violet, α-Fe—pink.

The XRD measurements were carried out at room temperature for all as-milled powders. The XRD patterns collected for all specimens are depicted in Figure 1. The subsequent broadening of diffraction peaks is noticed with the increase in milling time due to the reduction in crystallite size. It is worth mentioning that in XRD patterns, there is no evidence of other impurity traces, namely, from the ZrO_2_ milling media or samarium oxide. The observed broadening of diffraction peaks is associated with the gradual amorphization upon the duration of the grinding process manifested mainly as a significant decrease in crystallite size. As the next effect of mechanosynthesis, visible differences in the peak intensity are observed due to the separation of various crystal phases, the shape and size effects and the varied texture related to different orientations of the crystallites in the grains/particles.

**Figure 2:**
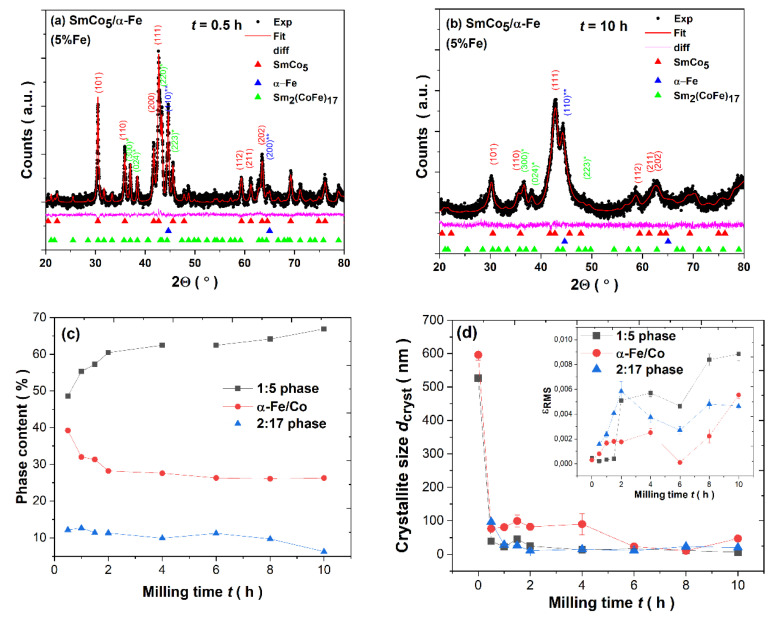
(**a**) Rietveld refinement of XRD pattern for SmCo_5_/α-Fe (5%wt. of α-Fe) powders milled for (**a**) *t* = 0.5 h; (**b**) *t* = 10 h. (**c**) The phase content of 1:5, α-Fe/Co and 2:17 phases, and (**d**) variation in crystallite size *d_cryst_* and lattice strain ε_RMS_ (insert) over *t* milling time.

The dominant 1:5 hexagonal CaCu_5_, space group P6/mmm, crystal phase is evidenced in all samples. Obviously, the presence of cubic α-Fe/Co as the composite component is evident. In addition, the 2:17 rhombohedral Th_2_Zn_17_ phase, space group hR57, is formed. The phase content was determined based on Rietveld refinement of diffraction patterns. The evolution of unit cell parameters determined based on crystal structure refinement is presented in Table 1. Examples of refined XRD patterns for 0.5 h- and 10 h-milled samples are presented in Figure 2a,b. Other refined patterns are shown in Figure S1a–f, while refinement factors are added into Table S1 in the Supplementary Materials. For the short-milled specimen, the lattice parameters of the main 1:5 phase equal a = 4.996 Å and c = 3.976 Å. The prolonged milling up to 10 h is reflected in the slight expansion of the unit cell a = 5.000 Å and c = 4.083 Å, also proved by the increase within a volume of the unit cell from 85.379 (0.5 h) to 88.410 Å^3^ (10 h). The observed phenomenon can be caused by the Co/Fe interdiffusion process associated with the evident difference in the atomic radii of both constituents (Co—1.25 Å, Fe—1.26 Å) and separation of the additional 2:17 phase. However, the influence of gradual amorphization and reduction in crystallite size cannot be neglected. It is accompanied by a reduction in α-Fe and 2:17 contents. Comparing to t = 0.5 h, where the estimated phase content is about 1:5—48.63%, α-Fe—39.22%, and 2:17—12.15%, after t = 10 h of synthesis, such relation is changed into 1:5—66.91%, α-Fe—26.28%, and 2:17—6.28%. Thus, the analysis of the phase content points to the significant increase in the 1:5 phase of about 18% (see Figure 2c).

The presence of an additional 2:17 phase was already observed for other SmCo_5_/α-Fe compounds with different Fe contents [22,48,49,50,51,52,53,54,55]. To see all numerical data for phase contents, please refer to Table S1 in the Supplementary Materials. The application of HEBM as a method of composite synthesis over the selected milling time t has a significant influence on the microstructure, demonstrated as, e.g., by the gradual decrease in the average crystallite size (*d_cryst_*) from about 526 (bulk SmCo_5_ compound) and 596.47 (α-Fe) to about 6.3 (SmCo_5_) and 47.19 nm (α-Fe), respectively (see Figure 2d). For more details, see Table S2 in the Supplementary Materials.

As it has been evidenced so far for other ball-milled intermetallics [16,17,32,33,34,35,36,37,38,39,40,41,58,59], the *d_cryst_* is significantly reduced in the first stages of mechanical synthesis (here, it is noted just after 0.5 h) and further grinding is reflected only in its slight variation. As it was evidenced by, e.g., Rama Rao et al. [53], the crystallite size for the same quantity of Fe is significantly reduced after 20 h of milling, starting from about 30 (4 h) to 5 nm (20 h). Further milling up to 50 h exhibits only a slight change in such parameter. Similar behavior was also noticed for 10%wt. Fe. The rapid decrease in *d_cryst_* during the first stages and its further subtle difference is an effect of the change within the deformation mechanism from plastic deformation to grain boundaries sliding, and the time point at which such deformation occurs is dependent not only on the milling parameters but also on the milled material. Please note that the final size of crystallites is also related to the duration of the synthesis process as well as being determined by the melting point, e.g., the final size after 100 h was found to be about 8 and 14 nm for individual components Fe (melting point at 1535 °C) and Co (melting point at 1495 °C), respectively [59,60].

The analysis of the lattice strain at 10 h of pulverization indicates its progressive increase (see Figure 2d). Considerable lattice straining is one of the critical effects for various ball-milled intermetallics [5,16,17,32,33,34,35,36,37,38,39,40,41] induced by the substantial lattice disorder generated during HEBM by different deformation mechanisms. The sintering of as-milled powders leads to an increase in crystallite size due to the crystallization process caused by the heat treatment [45]. The crystallite size is dependent on the sintering temperature, e.g., being raised from about 18 (973 K) to about 32 nm (1123 K) [45].

### 3.2. Microstructure: SEM and TEM

**Figure 3:**
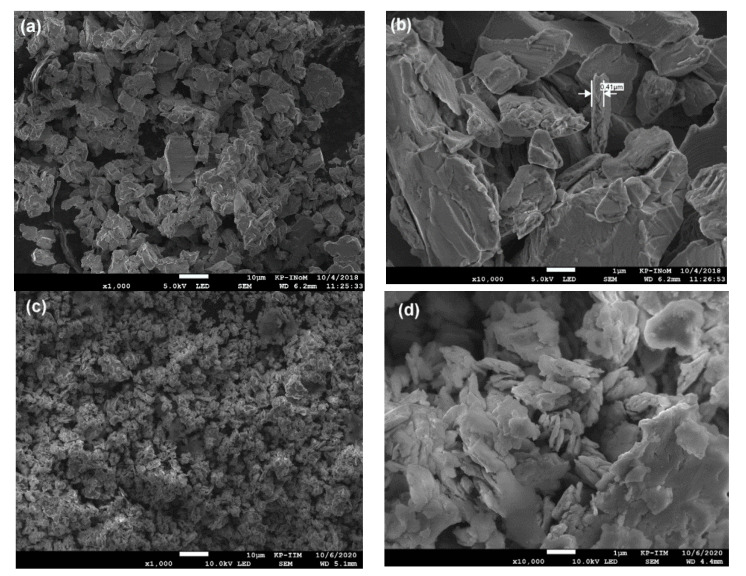
The microstructure of SmCo_5_/α-Fe (5%wt. of α-Fe) milled for 0.5 h (**a**,**b**); 10 h (**c**,**d**).

By analyzing SEM images, the microstructural evolution over the pulverization process is noted (see Figure 3). As one may observe, all as-milled powders are inhomogeneous and composed of irregular particles, which tend to form flakes during a longer mechanosynthesis time. For more details, see Figure S2 in the Supplementary Materials. Thus, the shape and size of as-milled powders vary across grinding times, and at the final stage (*t* = 10 h), the average thickness of particles is even less than 200 nm (see Figure 3d). Nonetheless, by analyzing SEM images, one may observe larger particles and finer ones, mostly agglomerated due to a tendency to reduce their surface via coalescence. As we have already shown, the synthesis of inhomogeneous R-T-based intermetallics is typical for the HEBM process [16,17,32,33,34,35,36,37,38,39,40,41] regardless of the type of crystal structure in as-synthesized powders. Noticeably, the wet milling performed in DMF leads to the formation of irregular micro/nanoparticles, which tend to agglomerate depending on their sizes.

**Figure 4:**
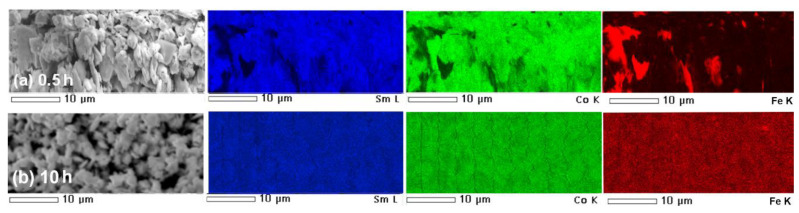
SEM energy-dispersive X-ray spectroscopy (EDS) elemental distribution maps measured for SmCo_5_/α-Fe (5%wt. of α-Fe) composites milled for (**a**) 0.5 and (**b**) 10 h.

The elemental map on the *t* = 0.5 h sample (see Figure 4a) indicates the non-homogenous distribution of the α-Fe powder in the SmCo_5_ matrix. One may notice regions with evident domination of iron powder, which is consistent with XRD data. The extended milling up to *t* = 10 h leads to significant homogenization of the as-milled powders. In this case, the elemental map (see Figure 4b) does not show regions with visible phase separation, characteristic for a shorter synthesis time. Thus, at the end of the synthesis, the as-milled powder is much more homogeneous.

**Figure 5:**
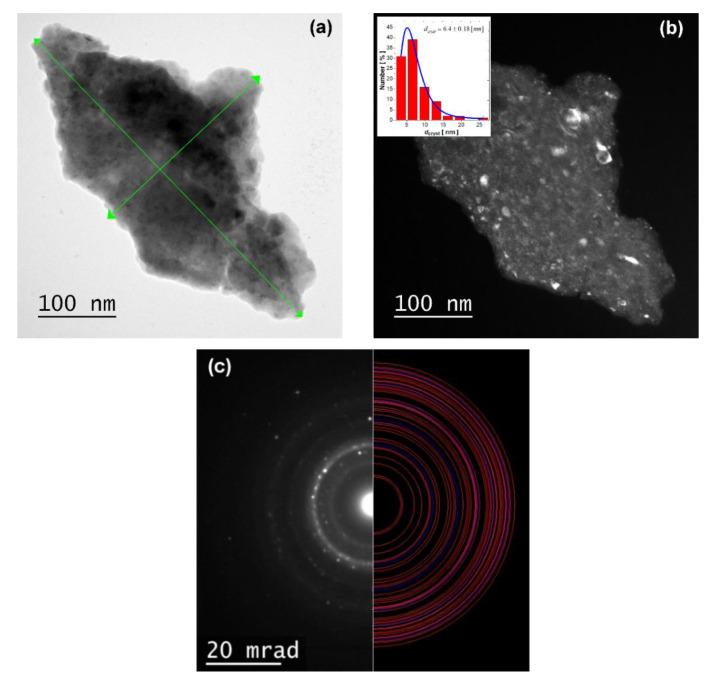
TEM micrographs for the SmCo_5_/α-Fe (5%wt. of α-Fe) sample milled for 10 h: (**a**) bright-field image (**b**); dark-field image with visible crystallites structure. Insert represents crystallite size distribution (CSD) histogram; (**c**) selected area electron diffraction (SAED) pattern indexed with CaCu_5_ (red lines) and α-Fe (blue lines) phases.

The TEM images of the SmCo_5_/α-Fe nanopowder ground for 10 h are shown in Figure 5. The samples milled for a shorter time were too thick for TEM imaging. The bright-field image (BF) exhibits a dark region of interest, pointing to a significant thickness of the studied material. The analysis depicting a region of interest reveals the irregular nanoflake particle with dimensions of 486 (length) and 254 nm (maximal width) as denoted by arrows. However, the detailed analysis of the dark-field image (DF) exhibits the presence of an irregular and non-homogeneous grained structure. The performed analysis of the crystallite size distribution by using the log-normal function allows for an estimate of the average crystallite size. Based on the crystallite size distribution (CSD) histogram, such parameter is estimated as <*d_cryst_*> ≈ 6.4 ± 0.18 nm. Such result is in good agreement with that obtained from the XRD pattern for the 1:5 phase, which dominates in the sample synthesized for *t* = 10 h. The selected area electron diffraction (SAED) pattern collected for the image depicted in Figure 5b was fully indexed according to the dominant hexagonal CaCu_5_ phase (red rings) and cubic α-Fe (blue rings) (see Figure 5c). The obtained electronogram is typical for polycrystalline and textured specimens. A certain degree of fortuitousness observed in the HEBM method TEM micrographs certainly confirms the polycrystalline heterogeneous nature of the SmCo5/α-Fe nanocomposite composed of inhomogeneously distributed and irregularly shaped crystallites.

### 3.3. Magnetic Properties

**Figure 6:**
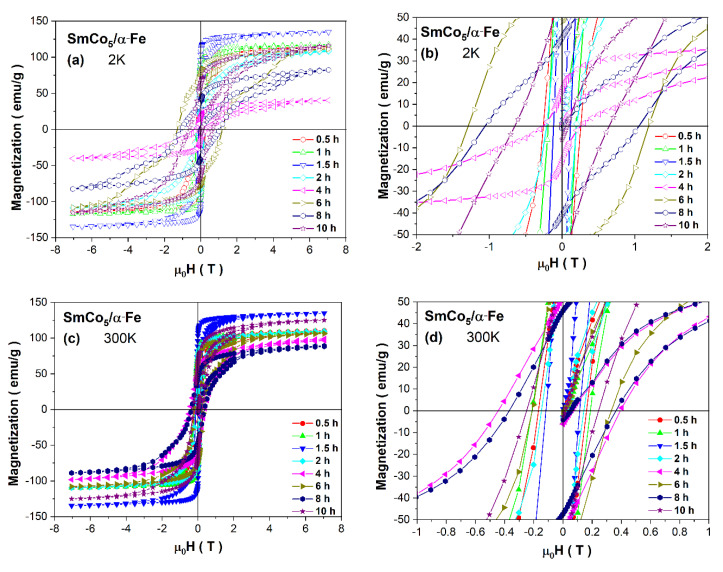
Hysteresis loops measured at 2 K, (**a**) and (**b**); and at 300 K, (**c**) and (**d**), for as-milled SmCo_5_/α-Fe (5%wt. of α-Fe) powders.

The hysteresis loops M(H) for all as-milled SmCo_5_/α-Fe (5%wt. of α-Fe) specimens are shown in Figure 6. By analyzing such curves at specific milling times, one may notice their deformation and the appearance of the characteristic kink. This may indicate the additional magnetic phase formed during HEBM, which is magnetically decoupled from the hard phase. Apart from that, it may also be associated with some other microstructural non-uniformities. Such behavior was already evidenced in other ball-milled intermetallics and is mostly related to the exchange spring mechanism [12,30,61,62,63]. In our case, we may observe such a deformation and characteristic kink especially at 2 K for t = 6 and 8 h, whereas at room temperature, such effect is not so clearly evident. Thus, at the low temperature for the middle t, the existence of a decoupling effect between hard SmCo_5_ and soft α-Fe phases seems to be prevailing. In addition, as one may observe, the magnetization at the external magnetic field (H_ext_) of 7 T is almost saturated in the low- and room-temperature regimes. However, the variation in M_7T_(t) dependence is non-linear, probably as an effect of the change within the particle size and shape and different exchange coupling between all magnetic phases present in nanopowders throughout the pulverization process. The observed non-linearity of M_7T_ (see Table 2) may also be due to possible agglomeration of finer particles, which tend to reduce their surface energy, especially under applying the external magnetic field. As demonstrated for other materials, the finer nanoparticles, nanoflakes, favor arrangements along an easy axis of magnetization, which is reflected as an evident increase in saturation magnetization [1,10,14,18,20,21,27,28,39].

The change in all magnetic parameters estimated from the hysteresis loops exhibits a non-linear variation (see Table 2). Generally, the observed modification of *H*c versus t is mainly related to the microstructural modification over the milling process and different phase contents. Usually, for ball-milled materials, in the first stages of synthesis, the coercivity increases due to effective continuous particle/grain size refinement. The further reduction within H_C_ values in HEBM is caused by the formation of flaked particles, crystallite refinement and a stronger exchange coupling between them [32,33,34,35,36,37,38,39,40,41]. In our case, such a mechanism is relatively sustained. Therefore, up to the middle synthesis time t, we can observe the gradual but non-linear increase in coercivity, which is obviously higher at 2 K than at room temperature, with its maximum of H_C_ = 1.24 T for *t* = 6 h. At room temperature, such a maximum is shifted to the shorter t and maxes for the sample milled for *t* = 4 h, where H_C_ = 0.42 T. The estimated coercivity is relatively low comparing to other ball-milled powders [4,5,6,7,8,9,10,11,12,13,14,15,16,17,18,19,20,21,22,23]. It is worth mentioning that other factors, e.g., an accumulation of defects created by grinding, larger shape anisotropy, variation within magnetic anisotropy, possible decay of the long-range magnetic order and gradual amorphization, may have a significant impact on H_C_(t) dependence.

The non-linear variation can also be noticed for the M_R_(t) dependence. However, in this case, the observed maximum value for this parameter is evident at *t* = 1.5 h (see Table 2) and is subsequently reduced for further milling. The observed non-linearity for remanence may be associated with (i) the change in the microstructure and consequently the shape anisotropy, (ii) the noticeable agglomeration of particles for the higher milling time, (iii) the variation within magnetic interactions between neighboring nanograins and modification within soft/hard magnetic coupling over milling or (iv) the change in the 1:5, α-Fe and 2:17 phase contents. In the research performed by Rama Rao et al. [53], it was shown that during milling, the coercivity is enhanced to about 7 kOe at 4 h and thereafter is significantly reduced for longer grinding to about 1 kOe noted for 54 h. Such a drastic decrease was explained by the authors as an evident effect of nanostructurization and amorphization of the 1:5 phase. Simultaneous modification within magnetization is caused by the probable disordering at the surface as an effect of the particle size reduction (initial decrease in magnetization) and recovery of Co moments preliminarily suppressed in the alloy from the elemental value due to localization by distortion (an increase in magnetization). The reduction in coercivity was also noted from nominal SmCo_5_ (4.6 kOe) to 10%wt. Fe (1.4 kOe). Further, an increase in the iron content in nanocomposites up to 35%wt leads to an ensuing H_C_ reduction [55]. One of the possibilities to improve coercivity is sintering the as-milled powders [45], e.g., from 973 (H_C_ ≈ 6 kOe) to 1073 K (H_C_ = 8.2 kOe), or optimization of milling parameters, e.g., under a magnetic field [22].

One of the utilizable tools for determining magnetic properties in nanomaterials is the analysis of the remanence-to-saturation (M_R_/M_S_) ratio, which is strongly dependent on the alteration within the particle size versus the grinding time [13,35,36,37,38,39,40,41,64]. As it was proposed by the Stoner–Wohlfarth (S–W) model, such ratio equals M_R_/M_S_ = 0.5 at T = 0 K [65] and is typical for randomly oriented single-domain nanoparticles. Such value can be exceeded due to the increase within the exchange coupling between neighboring nanograins caused by the deviation in magnetization from the easy axes. The enhanced parameter M_R_/M_S_ is generally observed for nanocrystalline hard magnets with a strong short-range exchange coupling between different nanograins [13,64,65,66]. The lowering of the M_R_/M_S_ << 0.5 ratio is usually demonstrated for non-interacting particles, which exhibit superparamagnetic behavior, generally due to the weak exchange coupling [67]. Bearing in mind the prediction of the S–W model, we may analyze the remanence-to-saturation ratio for the as-milled SmCo_5_/α-Fe powders with 5 wt. α-Fe%. In such an analysis, we took into account M_7T_ instead of M_S_. Thus, both at low temperature and room temperature, such ratios are relatively high and mostly higher or just below the limit predicted by the S–W model, which may point to relatively strong exchange coupling between nanograins slightly modified over pulverization. However, the observed phenomenon is also owing to multiple factors such as (i) a visible decrease within particle/crystallite size, (ii) an increased fraction of finer particles inclined at random orientation and incoherence in grain boundaries, (iii) an enhancement of the random anisotropy and (iv) destruction of the long-range magnetic order and its transformation into a short-range one during the milling process.

**Figure 7:**
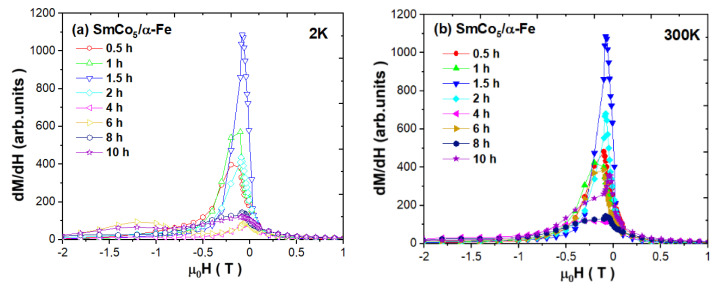
Derivatives of magnetization curves d*M*/d*H* versus *H* measured at (**a**) 2 and (**b**) 300 K for as-milled SmCo_5_/α-Fe (5%wt. of α-Fe) powders.

As one may notice, the hysteresis loops deteriorate with maximal deformation observed at *t* = 6 h (for 2 K) and *t* = 4 h (for 300 K). Such behavior is associated with the modification within the exchange coupling between hard and soft magnetic phases. One of the tools to analyze the hard and soft contributions of the composite magnetization process is a derivative of the remagnetization d*M*/d*H* curves versus H. For the as-milled SmCo_5_/α-Fe (5%wt. of α-Fe) powders, such curves are depicted in Figure 7. Generally, the area under curves d*M*/d*H* is related to the hard/soft magnetic species. As it was previously demonstrated by Pop et al. [52], the presence of only one thin peak placed at the low field is attributed to a narrow crystallite size distribution and stronger exchange coupling. The SmCo_5_/α-Fe composites with 20 wt.% milled for 6 and 8 h exhibit such a peak just below 0 T, and for such samples, coercivity is also very low, pointing to the vanishing of exchange coupling due to a reduction in the hard phase by milling. The subsequent annealing leads to a shift in the d*M*/d*H* maximum and a significant increase in coercivity.

Moreover, there is also a second maximum which is a proof for a mixture of uncoupled soft and hard phases [52]. In our case, at low temperature, we can see a relatively narrow peak just up to *t* = 2 h of the synthesis process, where we also note a significant increase in the 1:5 phase and a decrease in the α-Fe phase. For the higher milling time, the maximum is reduced and is shifted towards 0 T, which probably indicates that some Fe crystallites are not perfectly coupled. An additional maximum for *t* = 6 and 10 h is also observed, which can testify to the reduction in coupling and the significant crystallite size distribution. At room temperature, the variation in the d*M*/d*H* peaks is similar. It is worth mentioning that in both cases, the peaks’ width and long tails can indicate a significant percentage of crystallites which are not well coupled. Thus, the intensity of d*M*/d*H* peaks around H_C_ is relatively weak, pointing to the low contribution of a well-exchange-coupled composite, whereas the domination of the highest peak around small fields reveals the main contribution of a non-coupled soft magnetic phase. A visible shoulder at higher magnetic fields may correspond to a non-coupled hard magnetic phase. The interpretation of such curves is also rather unobvious due to the possible interdiffusion Co/Fe process.

The maximum energy product *(BH)_max_* as the maximum amount of magnetic energy stored in a magnet was estimated based on demagnetization curves which are shown in Figure S3 in the Supplementary Materials. As we may notice at the low temperature, such a parameter varies in a non-linear way and maxes at t = 6 h, where *(BH)_max_* = 84.7 kJ/m^3^, consistent with the change in coercivity. At room temperature, we observe the maximum *(BH)*_max_ = 35.1 kJ/m^3^ at *t* = 1 h. The estimated values are significantly lower than those recently published by Chakraborty et al. [57] for SmCo_5_/α-Fe with 10 wt.% Fe samples, where the maximum value of about *(BH)_max_* = 145 kJ/m^3^ was observed for specimens milled for 6 h and annealed at 510 °C.

Similarly, for the pure SmCo_5_ compound, the maximum energy product was determined as 231 kJ/m^3^ [2]. Previously, we have also shown that extended milling of SmCo_5_ is reflected in a drastic reduction in *(BH)_max_*, where the maximum of 129.7 kJ/m^3^ was observed at room temperature for the sample milled for *t* = 1 h [16,17,54]. Thus, the addition of 5 wt.% Fe leads to a significant reduction in such a parameter, probably due to the weak exchange coupling between the hard and soft phases of various grain sizes. As it was already published, the sintering of as-milled powders for different sintering temperatures leads to an improved *(BH)_max_* parameter up to almost 278 kJ/m^3^ for 1073 K, which is an effect of exchange coupling enhancement [45]. The modification within the maximum energy product can also be tuned by the change in the iron content combined with heat treatment [55]. The optimal addition of such a soft phase was estimated in the range of 20–30%wt., where *(BH)_max_* attains the maximum value of about 105.8 kJ/m^3^ for 25%wt. and an annealing temperature of 873 K. A slightly lower value of 84 kJ/m^3^ was found by Pop et al. [52] for the sample with 20%wt. milled for 8 h and annealed at 550 °C for 1.5 h. Thus, optimization of the iron content in nanocomposites followed by the optimization of synthesis parameters is crucial for enhancing the maximum energy product.

The magnetic anisotropy constants at different temperatures and milling times may be estimated from the approach to the saturation region by using the empirical law

(1) $$M\left(H\right)=M_{S}\left(1-\frac{a}{\sqrt{H}}-\frac{b}{H^{2}}\right)+\chi_{p}H$$

where *M(H)* is the magnetization measured at the external magnetic field, and *M_S_* is the saturation magnetization. The $a/\sqrt{H}$ term represents the strain field around dislocations by Brown and non-magnetic inclusions in voids by Néel [37], so it can be associated with point-like defects and intrinsic magnetostatic fluctuations, in which both, in nanocompounds, are strongly dependent on the particle size. The second term $b/H^{2}$ represents various kinds of anisotropies in the cubic compound, e.g., magnetocrystalline anisotropy, shape anisotropy or strain anisotropy, and can be approximated by

(2) $$b\approx \frac{8}{105}{\left(\frac{K_{1}}{\mu_{o}M_{S}}\right)}^{2}$$

where *K*_1_ is a magnetocrystalline anisotropy as a component of the effective anisotropy K_eff_. The last term *χ_p_H* represents the magnetic paraprocess caused by the partial suppression of spin waves by an external applied magnetic field. The fitting of *M(H)* was performed in the field range of 1–7 T, as it is shown in Figure S4, and estimated values of *M_S_*, *a*, *b* and *χ_p_* are placed in Table S3 in the Supplementary Materials. The analysis of the *K*_1_ parameter (see Table 2) reveals its modification versus the milling time. Generally, a reduction in the grain size may increase the effective inter-grain exchange interaction and decrease the effective magnetic anisotropy. However, in this case, the variation in the *K*_1_ parameter as a component of the effective anisotropy does not follow such a trend. Most likely, it is not only an effect of the modification within exchange interactions and the decrease in grain size but also of (i) the influence of the particle shape modified over milling, (ii) a variation in the 1:5, 2:17 and α-Fe phase contents and (iii) the subsequent Co/Fe interdiffusion process. Therefore, the change in anisotropy versus the milling time, which is typical for inhomogeneous HEBM powders, might be rather a resultant effect of all aforementioned factors than a specific trend. Nonetheless, the variation in anisotropy observed by Le Breton et al. [40] based on Monte Carlo simulations revealed a linear increase in the anisotropy constant within a Sm−Co−Fe mixed interface confirming that Fe/Co interdiffusion can lead to an increase in the coercive field.

### 3.4. Electronic Structure: XPS

**Figure 8:**
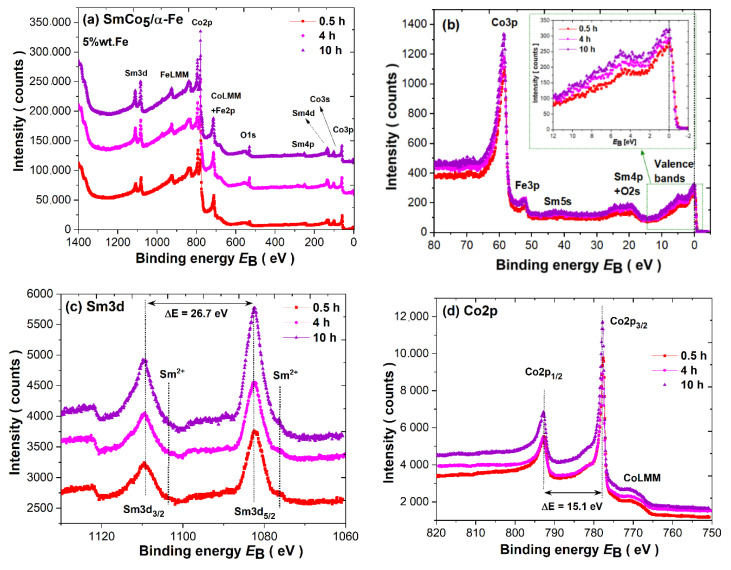

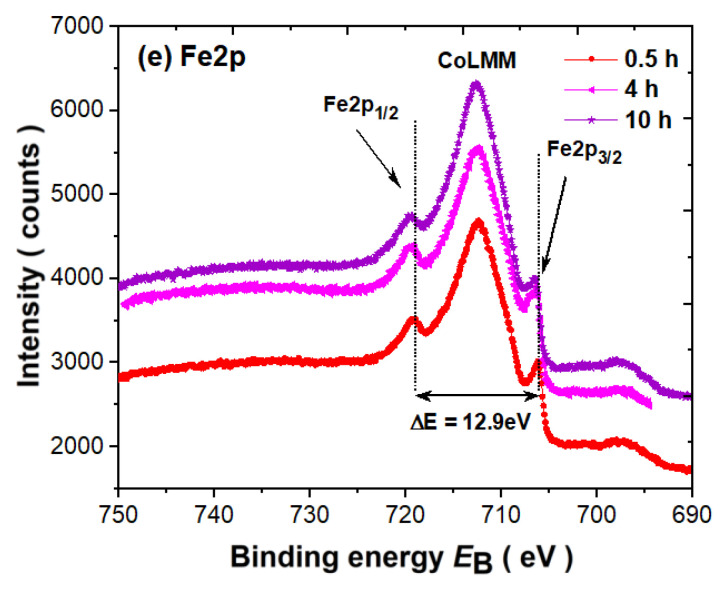
(**a**) Survey spectra; (**b**) valence bands; (**c**) Sm3d core level lines; (**d**) Co2p core level lines; and (**e**) Fe2p core level lines for as-milled SmCo_5_/α-Fe (5%wt. of α-Fe) powders.

The survey spectra measured after ion cleaning by an Ar^+^ beam for about 1 h are presented in Figure 8a. The main photoemission lines Sm3d, Co2p, Fe2p, Sm4p, Sm4d, Co3s and Co3p and the Auger CoLMM and FeLMM lines are denoted. There is no drastic change in all spectra collected for samples synthesized for *t* = 0.5, 4 and 10 h. The chemical composition estimated based on survey spectra acquired before and after ion cleaning is determined for comparison (see Table 3). The evident difference between both values is related to a significant quantity of surface impurities noted before using the Ar^+^ beam. After ion cleaning, the amount of C1s and O1s is significantly reduced. Note that the atomic concentration of iron and cobalt may be slightly disturbed due to the overlapping of 2p core lines with the Auger LMM lines.

The measured valence band spectra (VB) measured in a broad range together with the closely located core level lines are presented in Figure 8b. Around the binding energy of BE ≈ 58.6 eV, we may observe a Co3p line dominating over Fe3p located at about BE ≈ 52.1 eV. Sm5s states with significantly low intensity are observed just above 40 eV. In the range 30–16 eV, one may notice overlapped Sm4p and O2s states. The latter is observed despite surface ion cleaning as a residual oxygen impurity trace. The valence bands in the range −2 eV–12 eV are dominated by hybridized (Co/Fe)3d states with a typical 3d spectrum in the vicinity of the Fermi level (E_F_). The cusp around 5.2 eV is typical for trivalent Sm^3+^(4f) states. The divalent Sm^2+^(4f) states are overlapped with 3d and therefore, due to the domination of Co/Fe, its contribution can be barely noticed. The increase in the intensity of states in VB in relation to the t milling time is evident (see insert into Figure 8b) and may be related to the change within magnetic properties and the increase in the 1:5 phase content.

The Sm3d spectra reveal splitting due to a spin–orbit interaction of ΔE = 26.7 eV into 3d_5/2_ (BE ≈ 1082.6eV) and 3d_3/2_ (BE ≈ 1109.3 eV) lines. Such parameters are typical for metallic Sm^3+^ [68,69,70,71]. Nonetheless, below the main line, one may observe additional components visible as a shoulder with lower binging energy and lower intensity at BE ≈ 1076.1 and ≈ 1103.3 eV, respectively. Both lines are typical for Sm^2+^ states. Thus, the Sm3d spectrum consists of two components related to Sm^3+^ and Sm^2+^. The divalent Sm3d_5/2_ component associated with the 4f^6^ configuration is usually observed between 1070.0 and 1080.0 eV and the trivalent Sm3d_5/2_ (4f^5^) component is usually observed between 1080.0 and 1090.0 eV [68,69,70,71]. We do not observe any noticeable variation in or sifting of samarium lines versus the milling time. Therefore, the noted mixed-valence state is independent of the synthesis duration.

The Co2p_3/2_ (BE ≈ 777.6 eV) and Co2p_1/2_ (BE ≈ 792.7 eV) core level spectra are plotted in Figure 8d. The L–S splitting is stable during sample processing and equals about ΔE ≈ 15.1 eV. The Fe2p lines (see Figure 8e) are split into Fe2p_3/2_ (BE ≈ 706.6 eV) and Fe2p_1/2_ (BE ≈ 719.5 eV), with a splitting parameter of about ΔE ≈ 12.9 eV typical for metallic iron. Obviously, the CoLMM Auger line as the dominant component is overlapped with Fe2p. Nonetheless, the Co2p, as well as Fe2p, lines are shifted to a higher BE of about 0.2 eV, and such energy shifts can be caused by: (i) a change in the local environment of Co atoms induced by the modification of the phase content throughout the synthesis duration and (ii) the influence of hybridization of the Co3d states with Sm 5d6s and Fe3d, as well as conduction states. Generally, such chemical shifts, when compared to the corresponding pure metals, reveal a charge transfer from the Sm rare-earth to (Co/Fe)3d band. The observed chemical shift is usually larger for Sm-based compounds due to the mixed valence of Sm ions [69,70].

Multiplet splitting of the transition metal 3s core-level photoemission spectra is generally used to determine the magnetic moment of the 3d atom [72,73,74,75,76,77,78,79]. Its value is related to the partial filling of the 3d shell. Therefore, due to the quenching of the orbital moment, the 3d moment is around 2S. Using a simple model [50,51,52,53,54], the splitting of 3s spectra with two final states in relation to uncompensated 3d states can be discussed. As it is known, the initial state may be described as ^n+1^L, while after photoionization, the double final state associated with the different arrangements of the 3d^N^ spin and 3s vacancy is defined as the ^n+2^L high-spin term (S + 1/2) (parallel arrangement) and the ^n^L low-spin term (S − 1/2) (antiparallel arrangement). Therefore, in the 3s photoemission spectra, one may notice two peaks, namely, the main peak with higher intensity (I_A_) and the satellite peak with lower intensity (I_B_), as equivalents of two final states with the ratio

I_B_(^n^L)/I_A_(^n+2^L) = S/(S + 1) (3)

where S represents the spin moment of the unpaired 3d electrons. The splitting parameter (ΔE) between both peaks determined from the 3s fitting is proportional to the exchange overlap integral of 3s and 3d shells (J_3s–3d_) based on the relation

ΔE = (2S + 1) J_3s–3d_ (4)

In addition, the magnetic moment of the 3*d* atom can be ascribed as

(5) $$\mu_{3d}=2\mu_{B}\sqrt{S\left(S+1\right)}$$

**Figure 9:**
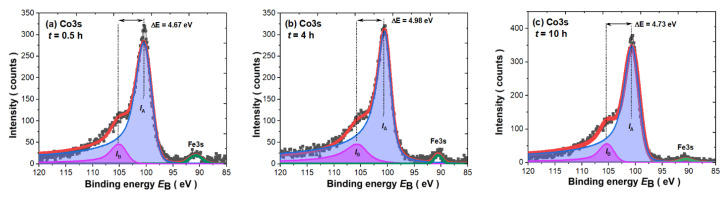
Co3s multiplet splitting determined in as-milled SmCo_5_/α-Fe powders with 5%wt. α-Fe content milled for: (**a**) *t* = 0.5 h; (**b**) *t* = 4 h; and (**c**) *t* = 10 h.

The results of the Co3s spectra fitting for powders milled for 0.5, 4 and 10 h are depicted in Figure 9. It is worth mentioning that the quantity of iron in the tested nanocomposites is too low to observe similar splitting to Co3s states, and that is why only one single peak around 90.5 eV can be noticed. Thus, all shown Co3s spectra were fitted by using an asymmetric Doniach–Sunjić (DS) type of line [55] with the same asymmetry parameter α for both I_A_ and I_B_ peaks as it was previously performed for other compounds [33,34,50,51,52,53,54]. All parameters obtained from the applied fitting procedure are presented in Table 4. As we have observed, the Co3s lines for all measured powders exhibit quite significant splitting. Laslo et al. [80] already performed Co3s analysis for the series of RCo_5_ compounds and the estimated ΔE parameter for the SmCo_5_ compound equaled 3.5 eV, which is significantly lower than that obtained by us. However, the presented results suggest that the authors did not use the typical asymmetric line shape in the fitting procedure, which is usually applied for 3s lines [72,73,74,75,76,77,78]. Nonetheless, the ΔE parameters obtained by us are close to those previously noted for pure Co, where they were found to be about 4.5–4.7 eV [72,73,74,75,79,80]. However, the value of the estimated cobalt magnetic moment is significantly lower than for pure Co (1.7 µ_B_) [79], probably due to the presence of α-Fe in the nanocomposite. Moreover, the increase in the milling time is reflected in a visible increase in (i) the Co magnetic moment and (ii) a slight increase in the 3s–3d exchange integral J_3s-3d_ values within the experimental uncertainty. The observed phenomena are related to the increase in the 1:5 phase and the reduction in α-Fe over the milling time as a consequence of the variation within the 3s–3d interaction due to hybridization effects within the (Co/Fe)3d band. The observed changes are also consistent with the VB, especially with the evident increase within the intensity of states at the Fermi level (see insert in Figure 8b).

## 4. Conclusions

In this study, we presented exchange-coupled SmCo_5_/α-Fe nanocomposites with 5%wt. of α-Fe content synthesized by wet HEBM for various periods up to 10 h. We demonstrated that the modification of the multiphase polycrystalline crystal structure over the milling process is reflected in: (i) the change in the 1:5, 2:17 and α-Fe phase contents, (ii) the decrease in crystallite size and the simultaneous increase in the lattice strain in the main hexagonal 1:5 phase and (iii) the slight expansion of the 1:5 unit cell, probably associated with the Co/Fe interdiffusion process. The microstructure of as-milled powders evolved from coarse grains/particles to finer nanoparticles/nanoflakes during the pulverization time. Generally, the magnetic properties of milled nanopowders are dependent on the microstructure. The analysis of hysteresis loops revealed the presence of exchange coupling between the hard and soft phases. The magnetic parameters vary in a non-linear way, probably due to size and shape effects, different phase contents and the subsequent modification within the exchange coupling strength. The maximum coercivity *H*_C_ above 1T was attained at low temperatures after *t* = 6–8 h of pulverization, but at room temperature, it was noted as about 0.4 T for *t* = 4 h. The maximum value of the *(BH)_max_* energy product of 35.1 kJ/m^3^ at room temperature was determined after *t* = 1 h of milling which, on account of the presence of the soft α-Fe phase with different exchange couplings, is significantly lower than that for the corresponding SmCo_5_ nanopowders. The electronic structure studies of valence bands, as well as core level lines, revealed the intermediate valence state of Sm ions in all measured as-milled samples associated with the 4*f^6^* (Sm^2+^) and 4*f*^5^ (Sm^3+^) samarium configurations. The analysis of the multiplet splitting of Co3*s* spectra indicated the evident increase in the Co magnetic moment and a slight increase in the *J_3s–3d_* exchange integral across the milling process, which is consistent with the increase in the 1:5 phase, the reduction in the α-Fe phase and the subsequent change within magnetic interactions in relation to hybridization effects within the (Co/Fe)3*d* band and Co/Fe interdiffusion process.

Summarizing, our approach shows a significant potential for cost-effective synthesis of SmCo_5_-based nanopowders by using a modified wet HEBM method. We are aware that the interrelated magnetism and microstructure can be strictly controlled by optimizing various HEBM parameters. Nevertheless, the approach adopted by us may provide a promising basis for the first step in the fabrication of exchange-coupled SmCo_5_/α-Fe nanocomposite magnets which can be, in the next step, annealed in order to enhance, e.g., the coercivity and the value of the maximum energy product.

## Figures and Tables

**Table 1:** Crystal structure parameters determined from Rietveld refinement of XRD pattern for SmCo_5_/α-Fe (5%wt. of α-Fe).

Milling Time t (h)	a (Å)	c (Å)	a/c	V (Å^3^)	a (Å)	V (Å^3^)	a (Å)	c (Å)	a/c	V (Å^3^)
	1:5				α-Fe		2:17			
0	4.977	3.980	1.251	85.379	2.8671	23.568	–	–	–	–
0.5	4.996	3.976	1.257	85.956	2.8672	23.571	8.406	12.223	0.688	514.364
1	4.922	3.975	1.256	85.787	2.8669	23.564	8.401	12.244	0.686	513.531
1.5	4.997	3.978	1.256	86.029	2.8691	23.618	8.422	12.213	0.690	517.350
2	5.001	3.975	1.258	86.108	2.8692	23.621	8.418	12.276	0.686	516.659
4	5.022	4.006	1.254	87.488	2.8818	23.932	8.459	12.323	0.686	524.196
6	5.002	3.978	1.258	86.198	2.8696	23.630	8.439	12.265	0.688	520.526
8	5.024	3.986	1.260	87.126	2.8797	23.881	8.480	12.273	0.691	528.194
10	5.000	4.083	1.225	88.410	2.8736	23.729	8.479	12.291	0.690	527.839

**Table 2:** Magnetic parameters for as-milled SmCo_5_/α-Fe (5%wt. of α-Fe) powders.

Milling Time t (h)	M_7T_ (emu/g)		µ_0_H_C_ (T)		M_R_ (emu/g)		M_R_/M_7T_		*(BH)*_max_ (kJ/m^3^)		K_1_ (erg/cm^3^) × 10^5^	
	2 K	300 K	2 K	300 K	2 K	300 K	2 K	300 K	2 K	300 K	2 K	300 K
0.5	114.2	107.9	0.26	0.16	87.4	69.1	0.77	0.64	46.0	24.2	2.75	5.62
1	117.2	108.7	0.19	0.2	90.5	76.7	0.77	0.71	33.9	35.1	2.31	5.05
1.5	134.8	135.0	0.16	0.12	103.7	103.1	0.77	0.76	24.3	25.6	4.74	4.76
2	108.2	109.4	0.23	0.15	73.7	71.8	0.68	0.66	28.2	21.9	5.59	6.67
4	40.3	96.7	0.26	0.42	16.2	51.9	0.40	0.54	3.1	27.4	3.11	10.34
6	111.5	106.1	1.24	0.29	78.4	69.5	0.70	0.66	84.7	32.9	6.63	7.87
8	82.5	88.3	1.06	0.35	40.9	46.9	0.60	0.53	24.5	20.7	8.35	8.35
10	115.7	125.1	0.65	0.25	63.2	66.9	0.55	0.53	40.4	23.3	9.78	9.84

**Table 3:** Chemical composition in at. % determined based on survey spectra for as-milled SmCo_5_/α-Fe (5%wt. of α-Fe).

Milling Time t (h)	Sample	O1s	C1s	Sm3d_5/2_	Fe2p_3/2_	Co2p_3/2_
0.5	surface	42.79	43.54	1.51	0.33	11.83
	etched	7.47	4.13	11.94	1.37	75.09
4	surface	39.84	47.86	1.38	0.18	10.73
	etched	17.92	10.37	13.94	0.10	57.67
10	surface	43.58	42.52	2.63	0.72	10.56
	etched	26.83	20.35	8.30	0.60	43.92

**Table 4:** Magnetic parameters for the SmCo_5_/α-Fe powders with 5%wt. α-Fe content obtained from Co*3s* fitting.

Milling Time t (h)	I_B_/I_A_	ΔE (eV)	α	S	2S	μ_Co_ (μ_B_)	J_3s-3d_ (eV)	χ^2^
0.5	0.167 ± 0.02	4.673 ± 0.03	0.23	0.200 ± 0.024	0.400	0.98 ± 0.5	3.34 ± 0.14	4.22
4	0.245 ± 0.02	4.980 ± 0.03	0.24	0.325 ± 0.053	0.650	1.31 ± 1.0	3.02 ± 0.21	2.09
10	0.159 ± 0.02	4.725 ± 0.03	0.23	0.189 ± 0.028	0.378	1.44 ± 0.4	3.43 ± 0.16	4.81

## Data Availability

The data presented in this study are available in Supplementary Materials.

## Supplementary Materials

The following are available online at https://www.mdpi.com/1996-1944/14/4/805/s1, Table S1: Phase content determined from Rietveld refinement of XRD pattern for SmCo_5_/α-Fe (5%wt. of α-Fe content) nanocomposite, Table S2: Crystallite size and lattice strain determined from Rietveld refinement of XRD pattern for SmCo5/α-Fe (5%wt. of α-Fe content) nanocomposite, Table S3: Magnetic parameters determined from hysteresis loops fitting for SmCo_5_/α-Fe (5%wt. of α-Fe content) nanocomposite, Figure S1: Rietveld refinement of XRD pattern for SmCo_5_/α-Fe (5%wt. of α-Fe content) for powders milled for (a) *t* = 1 h; (b) *t* = 1.5 h; (c) *t* = 2 h; (d) *t* = 4 h; (e) *t* = 6 h; (f) *t* = 8 h, Figure S2: The microstructure of SmCo_5_/α-Fe (5%wt. of α-Fe content) milled for: 1 (a) and (b); 1.5 (c) and (d); 2 (e) and (f); 6 (g) and (h); and 8 h (i) and (j), Figure S3: The relation of *H* versus *BH* with visible *(BH)_max_* estimated based on demagnetizing curves measured at (a) 2 and (b) 300 K for the SmCo_5_/α-Fe (5%wt. of α-Fe content) samples, Figure S4: The fitting of magnetization curves measured at (a) 2 and (b) 300 K for the SmCo_5_/α-Fe (5%wt. of α-Fe content) samples.
