# Supplementary material for: Correlation between Microstructure and Magnetism in Ball-Milled SmCo5/α-Fe (5%wt. α-Fe) Nanocomposite Magnets
Source: Materials (Basel). 2021 Feb 8;14(4):805. doi: 10.3390/ma14040805 (PMC7915708; doi:10.3390/ma14040805)
Supplement: Supplementary file 1 [file materials-14-00805-s001.zip › materials-1097857-supplementary.pdf]

materials

Supplementary materials

Correlation Between Microstructure and Magnetism in Ball-Milled $SmCo_5$/α-Fe (5%wt. α-Fe) Nanocomposite Magnets

Anna Bajorek [1,2*], Paweł Łopadczak [1,2], Krystian Prusik [2,3] and Maciej Zubko [2,3]

[1] A. Chełkowski Institute of Physics, University of Silesia in Katowice, 75 Pułku Piechoty 1, 41-500 Chorzów, Poland; p.lopadczak@gmail.com
[2] Silesian Center for Education and Interdisciplinary Research, University of Silesia in Katowice, 75 Pułku Piechoty 1A, 41-500 Chorzów, Poland; krystian.prusik@us.edu.pl (K.P.); maciej.zubko@us.edu.pl (M.Z.)
[3] Institute of Materials Science, University of Silesia in Katowice, 75 Pułku Piechoty 1A, 41-500 Chorzów, Poland
* Correspondence: anna.bajorek@us.edu.pl; Tel.: +48-32-349-7582

Table S1. Phase content and refinements factors determined from Rietveld refinement of XRD pattern for $SmCo_5$/α-Fe (5%wt. of α-Fe content) nanocomposite.

Milling Time t (h)	Sm(Co/Fe)$_5$ 1: 5	α-Fe	Sm_2(Co/Fe)$_{17}$ 2: 17	R_{wp}	R_B	R_{exp}
0.5	48.63	39.22	12.15	0.45616	0.34788	0.36525
1	55.33	32.01	12.66	0.45899	0.35581	0.37983
1.5	57.26	31.34	11.4	0.39556	0.31173	0.35519
2	60.45	28.2	11.35	0.36904	0.29046	0.3246
4	62.45	27.59	9.95	0.48229	0.30603	0.28105
6	62.46	26.29	11.25	0.45609	0.32526	0.33305
8	64.15	26.1	9.75	0.51087	0.33494	0.28133
10	66.91	26.28	6.28	0.32705	0.249	0.27554

Table S2. Crystallites size and lattice strain determined from Rietveld refinement of XRD pattern for $SmCo_5$/α-Fe (5%wt. of α-Fe content) nanocomposite.

Milling Time t (h)	Sm(Co/Fe)$_5$ 1: 5		α-Fe		Sm_2(Co/Fe)$_{17}$ 2: 17	
	d_{cryst} [nm]	ε_{RMS}	d_{cryst} [nm]	ε_{RMS}	d_{cryst} [nm]	ε_{RMS}
0	526.6 ± 3.508	4.45×10^{-4} ± $1,69 \times 10^{-5}$	596.469 ± 15.55	3.11×10^{-4} ± 6.7×10^{-6}	–	–
0.5	39.429 ±0.407	2.19×10^{-4} ± $1,89 \times 10^{-5}$	76.406 ± 5.337	8.03×10^{-4} ± 1.75×10^{-4}	96.269 ± 3.766	0.00157 ± 5.62×10^{-5}
1	22.885 ± 0.253	3.32×10^{-4} ± $4,31 \times 10^{-5}$	80.693 ± 1.171	0.00166 ± 2.05×10^{-4}	28.593 ± 0.252	0.00237 ± 2.26×10^{-4}
1.5	45.514 ± 3.732	4.02×10^{-4} ± $1,56 \times 10^{-4}$	99.389 ± 18.086	0.0018 ± 1.67×10^{-4}	26.387 ± 1.299	0.00407 ± 1.79×10^{-4}
2	24.997 ± 0.775	0.0051 ± $1,19 \times 10^{-4}$	81.761 ± 7.387	0.00176 ± 1.17×10^{-4}	10.804 ± 0.115	0.00584 ± 7.77×10^{-4}
4	13.444 ± 0.462	0.00571 ± $3,14 \times 10^{-4}$	89.773 ± 31.009	0.00251 ± 3.39×10^{-4}	15.202 ± 0.702	0.00375 ± 4.04×10^{-4}
6	11.45 ± 0.397	0.00465 ± $1,57 \times 10^{-4}$	23.436 ± 2.431	1.02×10^{-4} ± 7.9×10^{-5}	11.512 ± 0.297	0.0027 ± 3.02×10^{-4}
8	16.935 ± 0.74	0.00838 ± $4,67 \times 10^{-4}$	10.661 ± 0.924	0.00223 ± 5.42×10^{-4}	23.392 ± 2.194	0.00482 ± 4.06×10^{-4}

10	6.529 ± 0.125	0.00885 ± 5,45 × 10^{-4}	47.188 ± 0.126	0.00554 ± 2.98 × 10^{-4}	20.264 ± 0.726	0.00464 ± 2.07 × 10^{-4}

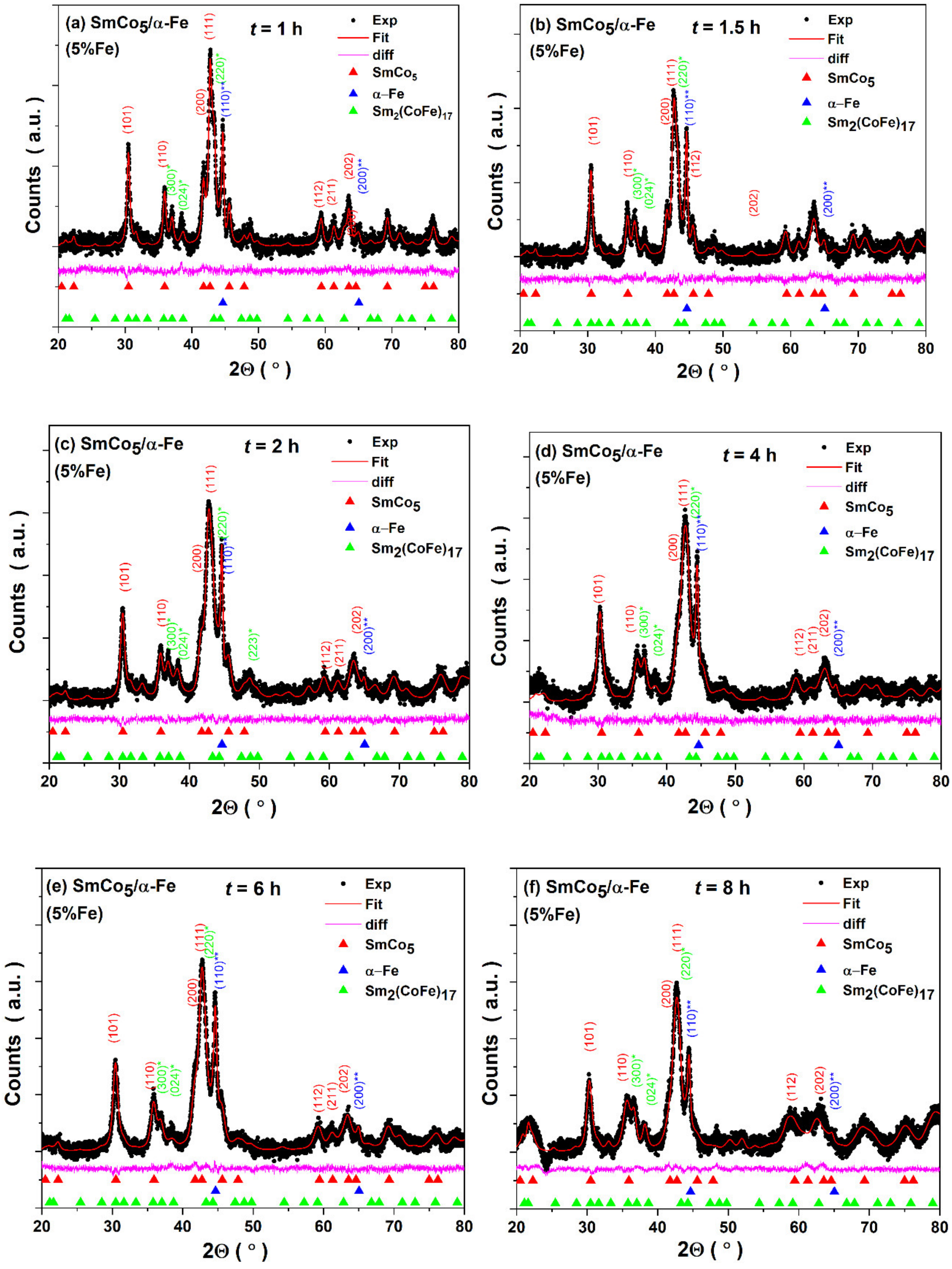

Figure S1. Rietveld refinement of XRD pattern for $SmCo_5$/α-Fe (5%wt. of α-Fe content) for powders milled for (**a**) *t* = 1 h; (**b**) *t* = 1.5 h; (**c**) *t* = 2 h; (**d**) *t* = 4 h; (**e**) *t* = 6 h; (**f**) *t* = 8 h.

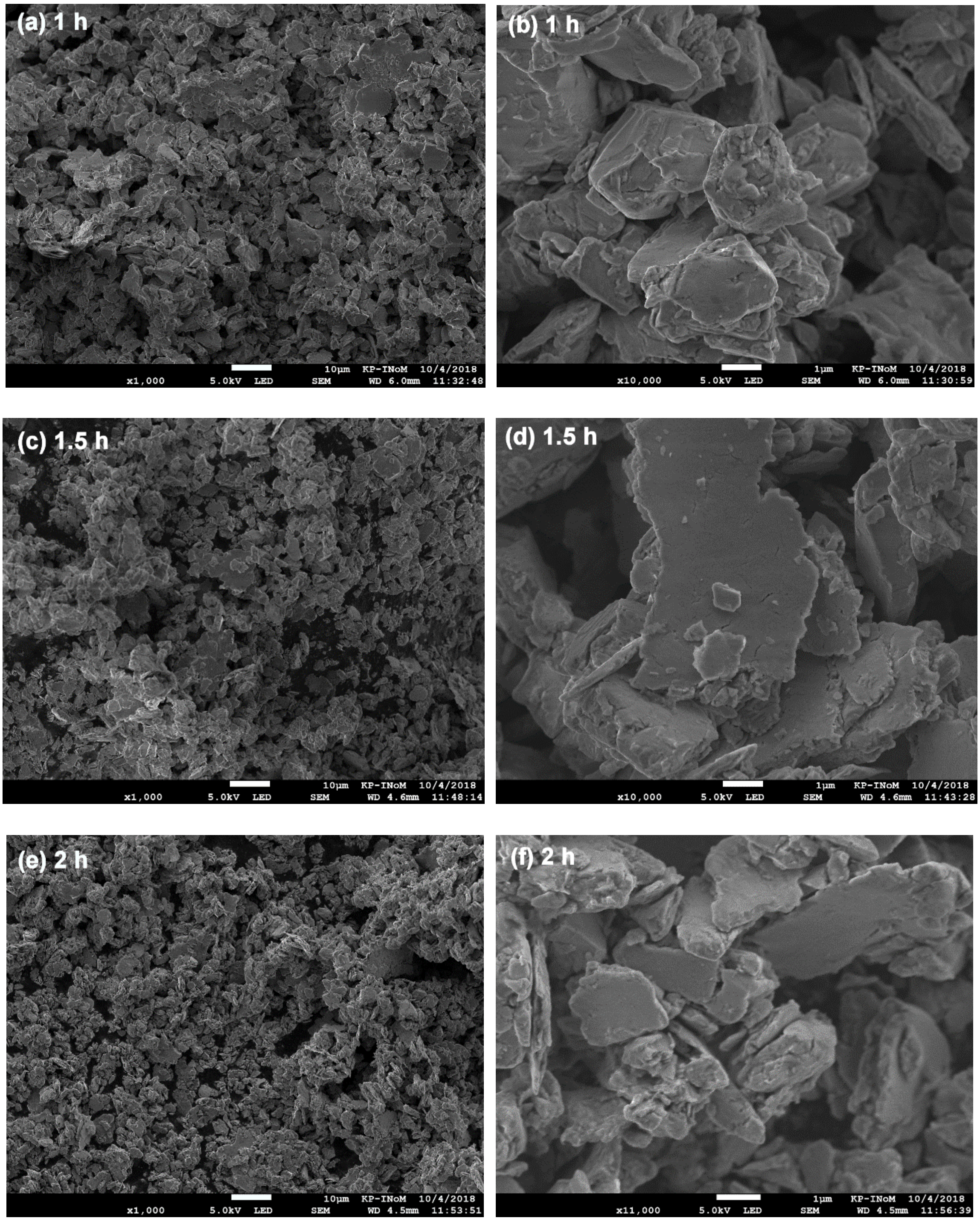
(a) 1 h
10μm KP-INoM 10/4/2018
x1,000 5.0kV LED SEM WD 6.0mm 11:32:48
(b) 1 h
1μm KP-INoM 10/4/2018
x10,000 5.0kV LED SEM WD 6.0mm 11:30:59
(c) 1.5 h
10μm KP-INoM 10/4/2018
x1,000 5.0kV LED SEM WD 4.6mm 11:48:14
(d) 1.5 h
1μm KP-INoM 10/4/2018
x10,000 5.0kV LED SEM WD 4.6mm 11:43:28
(e) 2 h
10μm KP-INoM 10/4/2018
x1,000 5.0kV LED SEM WD 4.5mm 11:53:51
(f) 2 h
1μm KP-INoM 10/4/2018
x11,000 5.0kV LED SEM WD 4.5mm 11:56:39

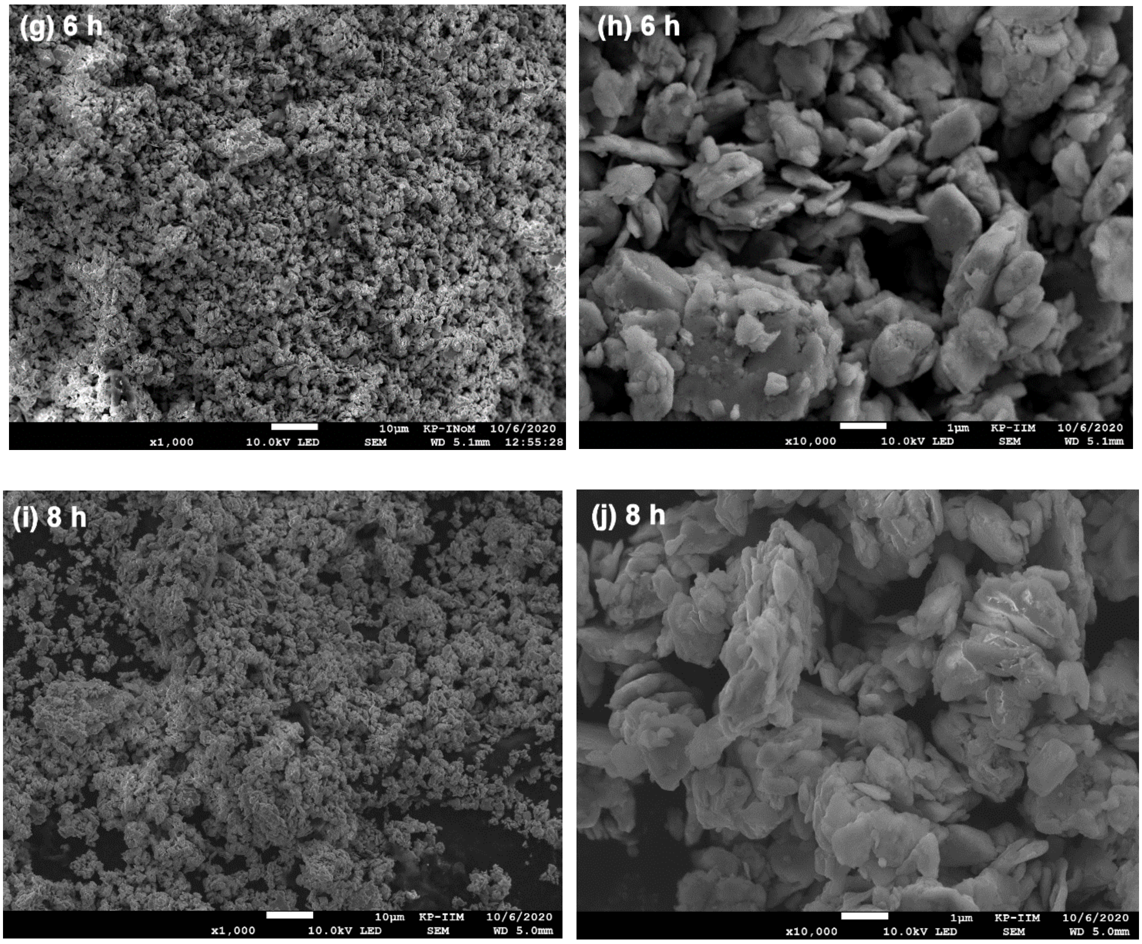

Figure S2. The microstructure of SmCo₅/α-Fe (5%wt. of α-Fe content) milled for: 1 h (**a**) and (**b**); 1.5 h (**c**) and (**d**); 2 h (**e**) and (**f**); 6 h (**g**) and (**h**); 8 h (**i**) and (**j**).

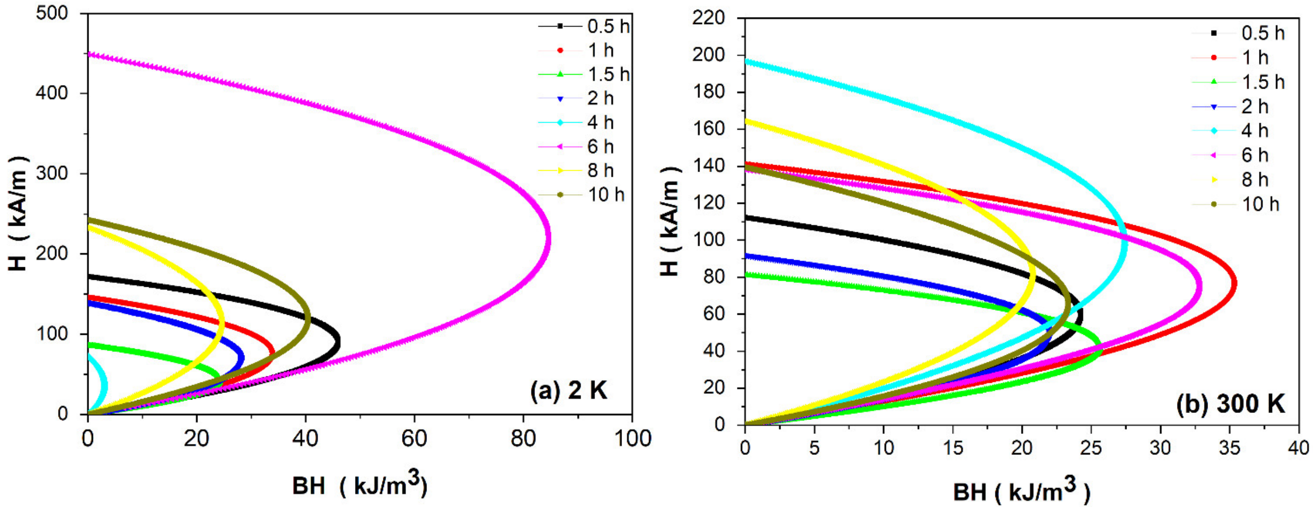

Figure S3. The relation of *H* versus *BH* with visible $(BH)_{max}$ estimated based on demagnetizing curves measured at (**a**) 2K and (**b**) 300 K for the $SmCo_5$/α-Fe (5%wt. of α-Fe content) samples.

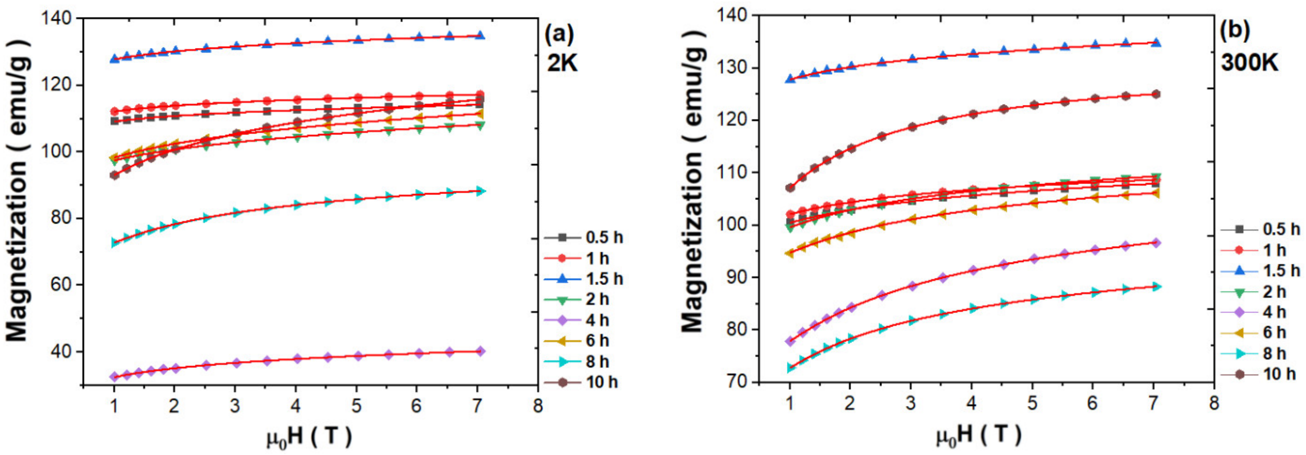

Figure S4. The fitting of magnetization curves measured at (**a**) 2K and (**b**) 300 K for the $SmCo_5$/α-Fe (5%wt. of α-Fe content) samples.

Table S3. Magnetic parameters determined from hysteresis loops fitting for $SmCo_5$/α-Fe. (5%wt. of α-Fe content) nanocomposite

t (h)	M_S (emu/g)		$a\ [\sqrt{kOe}]$		χ_P (emu/g Oe) × 10^{-3}		K_1 (erg/cm³) × 10^5	
	2K	300K	2K	300K	2K	300K	2K	300K
**0.5**	114.79 ± 0.59	112.69 ± 0.69	0.065 ± 0.011	0.168 ± 0.012	0.285 ± 0.033	0.212 ± 0.039	2.75 ± 0.69	5.62 ± 0.38
**1**	117.81 ± 0.46	112.98 ± 0.46	0.066 ± 0.008	0.143 ± 0.008	0.277 ± 0.063	0.160 ± 0.026	2.31 ± 0.59	5.05 ± 0.29
**1.5**	137.69 ± 0.69	137.35 ± 1.41	0.069 ± 0.012	0.099 ± 0.021	0.304 ± 0.079	0.304 ± 0.079	4.74 ± 0.98	4.76 ± 1.10
**2**	110.58 ± 0.61	116.61 ± 0.48	0.184 ± 0.008	0.226 ± 0.008	0.608 ± 0.022	0.198 ± 0.027	5.59 ± 0.21	6.67 ± 0.23
**4**	43.95 ± 0.20	114.24 ± 0.77	0.383 ± 0.008	0.510 ± 0.077	0.276 ± 0.011	0.196 ± 0.043	3.11 ± 0.08	10.34 ± 0.26
**6**	115.84 ± 0.39	115.93 ± 1.84	0.232 ± 0.006	0.291 ± 0.029	0.650 ± 0.222	0.173 ± 0.041	6.63 ± 0.20	7.87 ± 0.79
**8**	101.53 ± 0.61	102.68 ± 0.61	0.445 ± 0.010	0.453 ± 0.091	0.606 ± 0.034	0.106 ± 0.034	8.35 ± 0.23	8.35 ± 0.22
**10**	128.53 ± 0.76	145.58 ± 0.55	0.418 ± 0.010	0.370 ± 0.007	0.714 ± 0.043	0.349 ± 0.104	9.78 ± 0.08	9.84 ± 0.23
